# Effects of plyometric vs. traditional resistance training on kick performance and anaerobic power in elite taekwondo athletes

**DOI:** 10.3389/fspor.2026.1987540

**Published:** 2026-10-13

**Authors:** Feiyang Yu, Tan Tian

**Affiliations:** School of Sports Training, Wuhan Sports University, Wuhan, China

**Keywords:** combat sports, explosive strength, resistance training, roundhouse kick, stretch-shortening cycle, wingate test

## Abstract

**Background:**

Taekwondo requires high-velocity kicking actions and repeated explosive efforts, which depend heavily on lower-body power and stretch-shortening cycle function. Plyometric training may offer greater improvements in these demands than traditional heavy-resistance training, but direct evidence in elite taekwondo athletes is limited.

**Objective:**

This study compared the effects of 8 weeks of plyometric training and traditional resistance training on roundhouse kick performance, lower-body maximal strength, and anaerobic power in elite male taekwondo athletes.

**Methods:**

Twenty-four elite male taekwondo athletes (age 18–24 years, body mass 58–68 kg) were randomly assigned to either a plyometric training group (*n* = 12) or a traditional resistance training group (*n* = 12). Elite status was defined as holding a National First-Class Athlete certification and having won a provincial championship or placed in the top three at a national-level competition. Both groups trained twice per week for 8 weeks in addition to their regular taekwondo training. Before and after the intervention, participants were assessed for squat one-repetition maximum (1RM), roundhouse kick speed, kick power, 20-second continuous kick repetitions, countermovement jump (CMJ) height, reactive strength index (RSI), and Wingate peak and mean power. Data were analyzed using two-way repeated-measures ANOVA.

**Results:**

Significant group  ×  time interactions were observed for squat 1RM (F₁,₂₂ = 16.14, *p* = 0.001, partial *η*^2^ = 0.423), kick speed (F₁,₂₂ = 42.99, *p* < 0.001, partial *η*^2^ = 0.661), kick power (F₁,₂₂ = 35.46, *p* < 0.001, partial *η*^2^ = 0.617), kick repetitions (F₁,₂₂ = 9.54, *p* = 0.005, partial *η*^2^ = 0.303), CMJ height (F₁,₂₂ = 27.14, *p* < 0.001, partial *η*^2^ = 0.552), RSI (F₁,₂₂ = 52.90, *p* < 0.001, partial *η*^2^ = 0.706), and Wingate mean power (F₁,₂₂ = 125.56, *p* < 0.001, partial *η*^2^ = 0.851). The PT group showed greater pre-to-post improvements than the RT group in these variables: squat 1RM (+5.83 vs. + 3.00 kg; 95% CI for between-group difference in change: 1.37 to 4.30 kg), kick speed (+0.44 vs. + 0.07 m/s; 95% CI: 0.25 to 0.49 m/s), kick power (+142.75 vs. + 58.58 N; 95% CI: 54.86 to 113.48 N), kick repetitions (+1.92 vs. + 0.91; 95% CI: 0.33 to 1.67), CMJ height (+1.72 vs. + 0.65 cm; 95% CI: 0.63 to 1.49 cm), RSI (+0.09 vs. + 0.03; 95% CI: 0.04 to 0.08), and Wingate mean power (+64.83 vs. + 26.33 W; 95% CI: 31.37 to 45.63 W). No significant group  ×  time interaction was found for Wingate peak power (*p* = 0.460). At post-intervention, RSI and kick repetitions were significantly higher in the PT group than in the RT group (both *p* = 0.007), whereas other variables did not differ significantly between groups.

**Conclusion:**

Eight weeks of plyometric training resulted in greater pre-to-post improvements in roundhouse kick performance, reactive strength, and mean anaerobic power compared with traditional resistance training in elite male taekwondo athletes. These findings should not be interpreted as evidence that plyometric training is universally superior, because several outcomes did not differ significantly between groups at post-intervention.

## 1 Introduction

Taekwondo is a striking-based combat sport in which lower-limb actions dominate competitive performance (1). The roundhouse kick is the most frequently used technique in taekwondo competition, accounting for 44.7% of all trunk-level kicks, more than double the frequency of any other single technique (2). Its execution requires a rapid sequence of hip flexion, knee extension, and foot acceleration within a very short time window. Unlike movements that rely primarily on maximal force production, the roundhouse kick depends heavily on the ability to rapidly switch from eccentric loading to concentric drive in the support leg, a quality commonly referred to as stretch-shortening cycle function. For this reason, explosive lower-body capacity is considered a key determinant of taekwondo performance, particularly in lighter weight categories where movement speed plays a decisive role (3).

Plyometric training has been shown to improve vertical jump height, reactive strength, and short sprint performance across a range of athletic populations (4, 5). These adaptations are generally attributed to enhanced utilization of the stretch-shortening cycle, increased musculotendinous stiffness, and improved neural activation during rapid force production (6). Because these physiological changes align closely with the mechanical demands of taekwondo kicking, plyometric training has been proposed as a suitable method for improving kick-specific performance. However, direct evidence in elite taekwondo athletes remains limited, and the extent to which plyometric training improves roundhouse kick speed and force has not been clearly established. Furthermore, traditional high-resistance training is another common approach for developing lower-body strength in combat athletes, but its effects on high-velocity movements such as kicks are less consistent (7, 8). Some studies suggest that gains in maximal strength do not automatically transfer to explosive actions unless accompanied by movements performed at high speed (9). Given that taekwondo athletes must execute kicks at high velocities while managing body mass within specific weight divisions, the relative benefits of plyometric training and traditional resistance training for kick performance remain an open question.

Although plyometric training and traditional resistance training have each been studied in combat sports, few studies have directly compared their effects on kick-specific performance and anaerobic power in elite taekwondo athletes. In addition, most previous studies have focused on general physical capacities rather than sport-specific kicking performance. Therefore, the present study was designed to fill this gap by comparing the two training modes in a randomized controlled trial.

The present study therefore aimed to compare the effects of an 8-week plyometric training program and a traditional resistance training program on roundhouse kick performance, lower-body maximal strength, and anaerobic power in elite male taekwondo athletes. It was hypothesized that plyometric training would lead to greater improvements in kick speed, kick power, and repeated kick ability, as well as in CMJ height and RSI, compared with traditional resistance training. Traditional resistance training was expected to produce larger gains in squat 1RM. Both interventions were expected to improve Wingate anaerobic performance, with no strong directional hypothesis for between-group differences.

## 2 Methods

### 2.1 Study design

This study employed a randomized controlled trial design to compare the effects of 8 weeks of plyometric training (PT) and traditional resistance training (RT) on roundhouse kick performance, lower-body maximal strength, and anaerobic power in elite male taekwondo athletes. Participants were randomly assigned to either the PT group or the RT group. All outcome measures were assessed at baseline and after the 8-week intervention period. Post-intervention testing was completed 48–72 h after the final training session to minimize the confounding effects of acute fatigue or post-activation potentiation. All testing sessions were conducted at the same time of day (09:00–11:00) to control for diurnal variation in neuromuscular performance (10).

### 2.2 Participants

Twenty-four elite male taekwondo athletes were recruited from the men's taekwondo team of Wuhan Sports University. In this study, participants were classified as elite athletes based on the following criteria: (1) holding a National First-Class Athlete certification, and (2) having won a provincial championship or placed in the top three at a national-level competition. All participants met these criteria. Inclusion criteria were: (1) age between 18 and 23 years; (2) a minimum of 4 years of sport-specific taekwondo training; (3) current participation in national-level competitions; and (4) body mass within the 58–68 kg range. Exclusion criteria included any lower-limb injury in the previous 6 months, musculoskeletal disorders, or cardiovascular conditions that could compromise testing or training. Randomization was performed by an independent researcher using a computer-generated random sequence (https://www.random.org). Allocation was concealed using sequentially numbered, opaque, sealed envelopes. Participants were stratified by baseline roundhouse kick velocity and one-repetition maximum (1RM) squat strength, then randomly assigned in equal numbers to either the plyometric training (PT) group or the traditional resistance training (RT) group. Written informed consent was obtained from all participants, and the study was approved by the Medical Ethics Committee of Wuhan Sports University in accordance with the Declaration of Helsinki. Baseline characteristics of the two groups are presented in Table 1. All 24 participants completed the study and were included in the final analysis. A participant flow diagram is presented in Figure 1.

**Table 1:** Baseline characteristics of participants (mean ± SD).

Variable	PT (*n* = 12)	RT (*n* = 12)	F	p
Age (y)	20.3 ± 1.0	19.9 ± 1.2	0.57	0.457
Height (cm)	177.6 ± 3.1	176.9 ± 3.0	0.28	0.603
Body mass (kg)	62.2 ± 2.7	62.0 ± 3.2	0.02	0.890
Training experience (y)	4.7 ± 0.7	5.0 ± 0.8	1.23	0.278

PT, plyometric training; RT, traditional resistance training.

**Figure 1:**
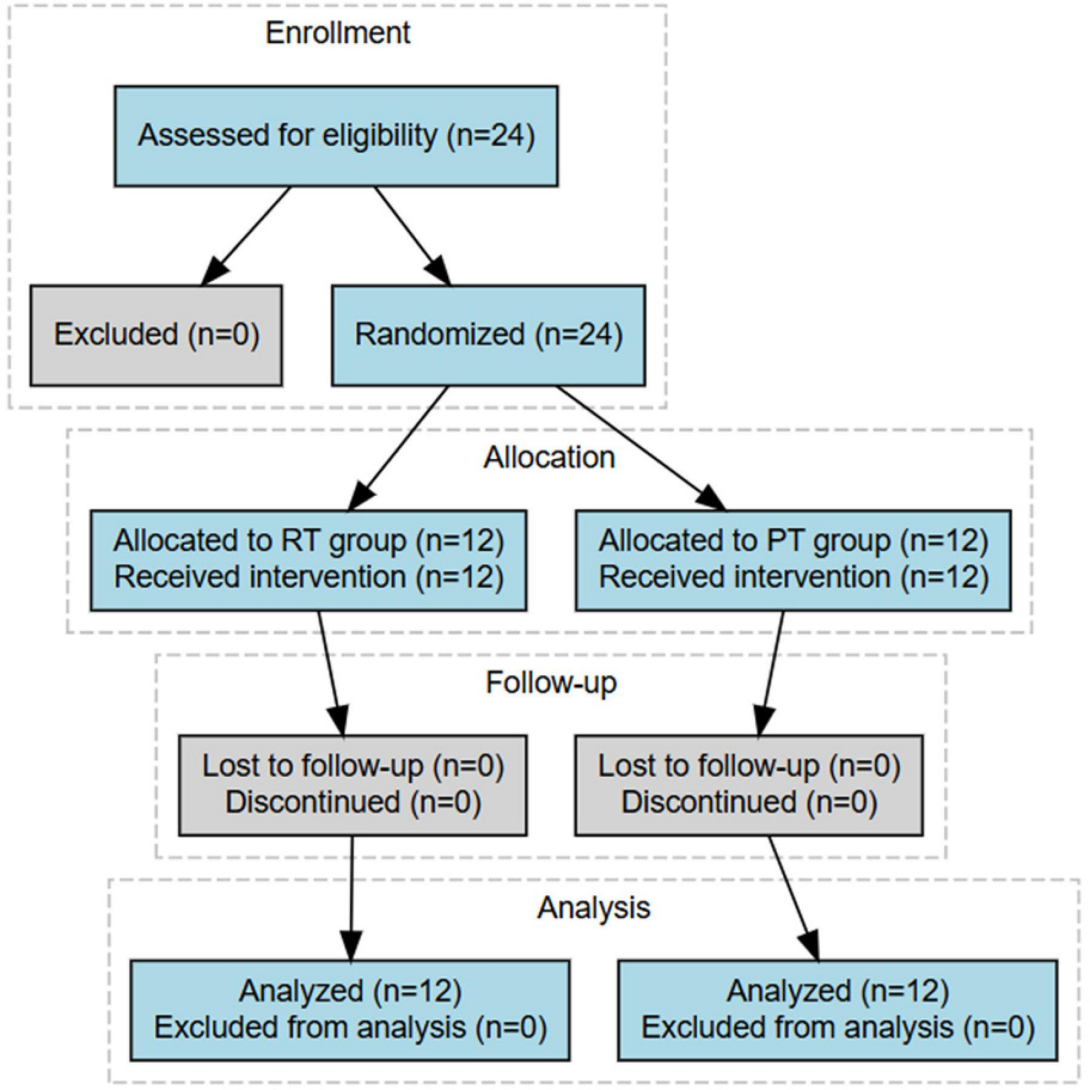
CONSORT flow diagram of participant recruitment, allocation, follow-up, and analysis. PT, plyometric training; RT, traditional resistance training. All 24 participants completed the study and were included in the final analysis.

### 2.3 Sample size estimation

The required sample size was estimated *a priori* using GPower software (version 3.1.9.7). A large effect size (Cohen's f = 0.40, partial *η*^2^ ≈ 0.14) was selected based on previous meta-analyses showing that plyometric training produces large improvements in vertical jump and kick performance in combat athletes (11). With *α* = 0.05 and statistical powe*r* = 0.80, the minimum sample size required was estimated to be 20 participants. To account for a potential dropout rate of approximately 15%, 24 participants were recruited, with 12 assigned to each group. **The following parameters were used in GPower: Test family = *F*-tests; Statistical test = ANOVA: Repeated measures, within-between interaction; Number of groups = 2; Number of measurements = 2; Correlation among repeated measures = 0.5; Nonsphericity correction *ε* = 1.**

### 2.4 Training interventions

Both groups continued their regular technical and tactical taekwondo training (∼10–12 h per week) throughout the study. Both groups completed the same technical and tactical taekwondo training program under the same coaching staff, with comparable weekly training volume. No additional lower-body conditioning was performed outside the study protocol. The intervention sessions replaced the usual physical conditioning component and were performed twice per week for 8 weeks, with at least 48 h between sessions.

Training adherence was recorded for each session and calculated as the number of completed sessions divided by the total number of scheduled sessions. Adherence was 100% in both the PT and RT groups. No participants dropped out, and no adverse events were reported during the intervention.

Session RPE was recorded after each training session using the CR-10 scale, and training load was calculated as session RPE multiplied by training duration. The mean session RPE was 7.2 ± 0.4 in the PT group and 7.4 ± 0.6 in the RT group, with no significant difference between groups (*p* > 0.05).

#### 2.4.1 Plyometric training group

The PT group completed a progressive plyometric training program lasting approximately 35–40 min per session, designed according to previously described progression models for explosive lower-body power (5, 12). Training was divided into four 2-week phases with increasing intensity and specificity (Table 2). Participants were instructed to perform all repetitions at maximal intended velocity, minimize ground contact time, and maintain proper landing mechanics. Rest intervals were 60–90 s between sets and 2–3 min between exercises. If movement quality deteriorated, intensity was reduced and additional rest was provided.

**Table 2:** Overview of the 8-week plyometric training program.

Phase	Weeks	Focus	Example exercises	Sets × Reps
1	1–2	Foundation	Submaximal vertical jumps, box jumps (30 cm), lateral jumps, alternating split squat jumps	3 × 6–8
2	3–4	Progression	Continuous hurdle jumps, single-leg hops, low-box depth jumps (30 cm), lateral bounds	3–4 × 6
3	5–6	Intensification	Depth jumps (40 cm), single-leg box jumps (20 cm), multi-directional hurdle jumps, depth jump to lateral bound	4 × 5–6
4	7–8	Specificity	Depth jump to kick-specific drive, single-leg hop to kick drive, continuous lateral jumps, depth jump to sprint	3–4 × 5–8

#### 2.4.2 Traditional resistance training group

The RT group performed lower-body heavy-resistance exercises: barbell back squat (4 × 5 at 80%–85% 1RM), conventional deadlift (3 × 5 at 75%–80% 1RM), Bulgarian split squat (3 × 6 per leg at 70%–75% 1RM), and standing calf raise (3 × 10). Rest intervals were 2–3 min between sets and 3 min between exercises. Training load was increased by 2.5–5 kg when all prescribed repetitions were completed with proper technique. This protocol was selected to emphasize maximal strength development, consistent with guidelines for resistance training in combat athletes (13).

### 2.5 Testing procedures

All tests were conducted in a climate-controlled laboratory (20–22 °C). Participants refrained from strenuous exercise for 48 h and from caffeine and alcohol for 24 h before testing. A standardized warm-up consisting of 5 min cycling at low intensity, dynamic stretching, and submaximal practice trials preceded each testing session. The testing order was: countermovement jump (CMJ), reactive strength index (RSI), 1RM back squat, single roundhouse kick, 20-s continuous kick test, and Wingate test. At least 30 min rest separated the strength and kick tests to minimize fatigue carryover. The Wingate test was always performed last due to its maximal nature. Prior to the formal testing, all participants completed a familiarization session and test-retest reliability was assessed under identical conditions; the reliability of all measurements was acceptable.

#### 2.5.1 One-repetition maximum back squat

Lower-body maximal strength was measured using the 1RM back squat (13). After warm-up sets at 50% (8 repetitions) and 70% (3 repetitions) of estimated 1RM, single repetitions were performed with progressively increasing loads until the participant could not complete the lift with proper technique. A maximum of five attempts was allowed, with 5 min rest between attempts. A linear position transducer (T-Force System, Ergotech, Murcia, Spain) was used to monitor barbell velocity and assist in judging repetition quality and proximity to maximal load.

#### 2.5.2 Countermovement jump and reactive strength index

CMJ height and RSI were assessed using a force plate (Kistler 9260AA, Winterthur, Switzerland) sampling at 1,000 Hz (14). For CMJ, participants performed three maximal jumps with hands placed on the hips, separated by 30 s. Jump height was calculated from flight time, and the average of three trials was used. For RSI, participants performed drop jumps from a 30 cm box, landing with both feet on the force plate and immediately jumping upward as quickly as possible. RSI was calculated as jump height (m) divided by ground contact time (s) (15). Three maximal trials were completed with 60 s rest, and the best value was retained.

#### 2.5.3 Kick performance

Roundhouse kick performance was assessed using a wall-mounted force plate (Kistler 9260AA) and a three-dimensional motion capture system (Vicon, Oxford Metrics, UK; eight cameras, 1,000 Hz) (16). The force plate was mounted vertically at shoulder height and covered with a standardized taekwondo striking pad to simulate a realistic target. Reflective markers were placed on the dorsum of the foot and the lateral malleolus of the kicking leg. After two submaximal practice kicks, participants performed three maximal-effort roundhouse kicks aimed at the center of the force plate, with 60 s rest between kicks. The trial with the highest peak force was selected for analysis. Peak force was recorded as the maximum value from the force plate. Peak velocity was determined as the maximum resultant velocity of the foot marker from the Vicon data. Impulse was calculated as the integral of the force–time curve during the contact period, starting when force exceeded 5 N. A 20-s continuous roundhouse kick test was also performed. Participants stood facing the force plate at the same standardized distance and, after a 3-s countdown, performed as many maximal-effort roundhouse kicks as possible within 20 s. Total kick count was recorded by two independent observers, and mean kick velocity was obtained from the Vicon foot marker data. Standardized verbal encouragement was provided every 2 s.

#### 2.5.4 Wingate anaerobic test

Anaerobic capacity was evaluated using a 30-s Wingate test on a calibrated electromagnetically braked cycle ergometer (Monark 894E, Varberg, Sweden) (17). Following a 5-min warm-up at 60–70 rpm that included two 4-s sprints, participants performed a maximal 30-s effort against a resistance of 0.075 kg/kg body mass. Peak power (highest 5-s average) and mean power (30-s average) were recorded.

### 2.6 Statistical analysis

Because the within-subject factor (time) had only two levels, the assumption of sphericity was automatically satisfied, and no correction was necessary.

Data are presented as mean ± standard deviation. Normality was assessed using the Shapiro–Wilk test, and baseline differences between groups were examined using independent-samples t-tests or Mann–Whitney U tests, as appropriate. For each outcome variable, a two-way repeated-measures analysis of variance (ANOVA) was conducted with group (PT vs. RT) as the between-subject factor and time (pre vs. post) as the within-subject factor. The primary interest was the group  ×  time interaction. When a significant interaction was observed, simple effects analyses were performed, and all pairwise comparisons were adjusted using the Bonferroni method. Effect sizes were calculated as partial eta squared (*η*^2^_p), with 0.01, 0.06, and 0.14 interpreted as small, medium, and large effects, respectively (18). Between-group differences in change scores were also reported with 95% confidence intervals. Statistical significance was set at *p* < 0.05. All analyses were performed using SPSS version 27.0 (IBM Corp., Armonk, NY, USA).

## 3 Results

### 3.1 Baseline characteristics

Baseline values for all outcome measures are presented in Table 3. There were no significant differences between the PT and RT groups in squat 1RM, kick speed, kick power, kick repetitions, CMJ height, RSI, Wingate peak power, or Wingate mean power at baseline (all *p* > 0.05), indicating that the two groups were comparable before the intervention.

**Table 3:** Baseline (pre-intervention) values of outcome measures (mean ± SD).

Variable	PT (*n* = 12)	RT (*n* = 12)	P
Squat 1RM Pre (kg)	89.42 ± 8.66	87.75 ± 9.87	0.664
Kick speed Pre (m/s)	11.10 ± 0.84	10.93 ± 0.99	0.649
Kick power Pre (N)	3,173.17 ± 159.31	3,168.50 ± 176.41	0.946
Kick repetitions Pre (n/20s)	14.75 ± 1.22	14.67 ± 1.07	0.860
CMJ height Pre (cm)	41.31 ± 1.33	40.34 ± 4.83	0.512
RSI Pre	1.34 ± 0.07	1.34 ± 0.06	1.000
Wingate peak power Pre (W)	736.42 ± 57.35	742.75 ± 57.64	0.790
Wingate mean power Pre (W)	534.33 ± 46.78	541.75 ± 56.17	0.729

PT, plyometric training; RT, traditional resistance training; CMJ, countermovement jump; RSI, reactive strength index. *p* values were obtained using independent-samples t-tests comparing baseline values between groups. For CMJ height, the *p* value is reported from the t-test that does not assume equal variances because Levene's test indicated unequal variances (*p* < 0.05); all other *p* values are from the equal variances assumed row.

### 3.2 Lower-body maximal strength

A significant group  ×  time interaction was observed for squat 1RM (F₁,₂₂ = 16.137, *p* = 0.001, partial *η*^2^ = 0.423). As shown in Figure 2C, the PT group increased from 89.42 ± 8.66 kg to 95.25 ± 7.83 kg, corresponding to a mean change of +5.83 kg (95% CI: 4.80 to 6.87 kg), while the RT group increased from 87.75 ± 9.87 kg to 90.75 ± 8.82 kg, with a mean change of +3.00 kg (95% CI: 1.97 to 4.03 kg). Both groups improved significantly from pre- to post-intervention (both *p* < 0.001). However, no significant between-group difference was detected at baseline (*p* = 0.664) or after training (*p* = 0.200). These results indicate that the significant group  ×  time interaction reflects a greater pre-to-post change in the PT group compared with the RT group, rather than a significant between-group difference at the post-intervention assessment (Table 4).

**Figure 2:**
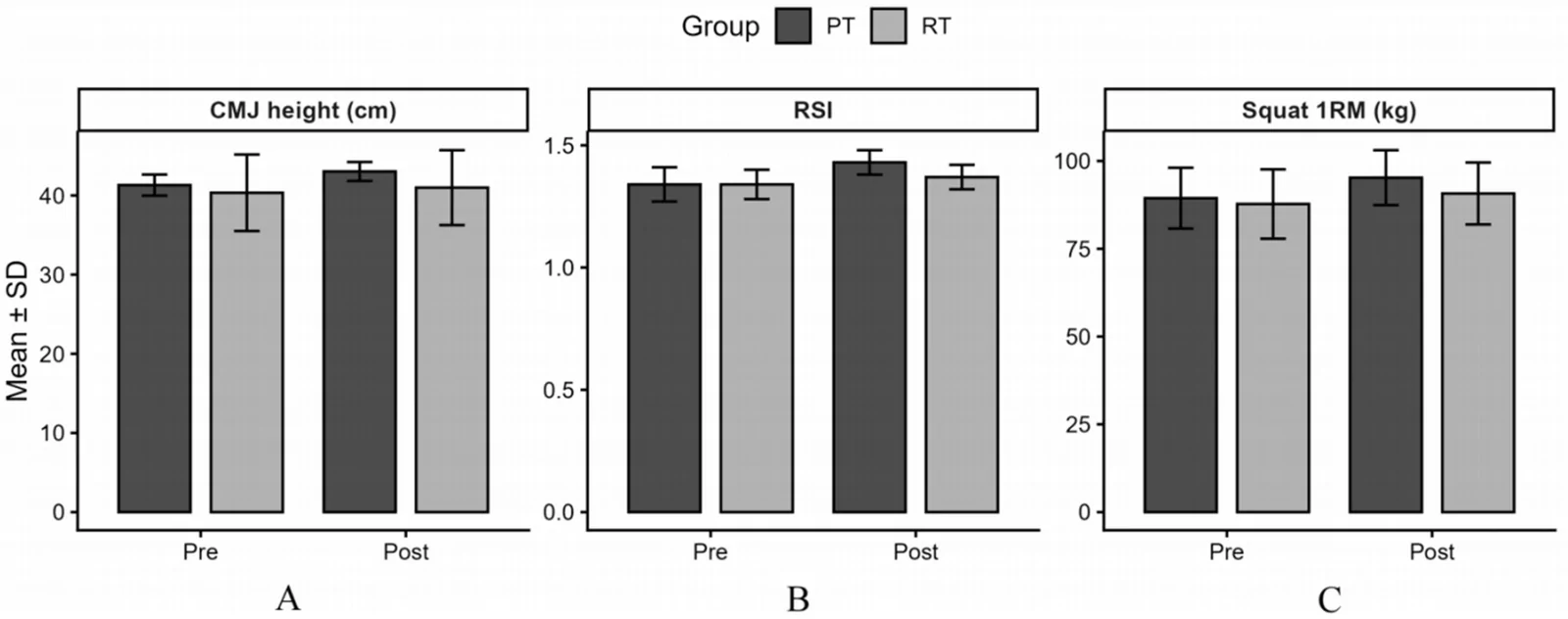
Changes in **(A)** countermovement jump (CMJ) height, **(B)** reactive strength index (RSI), and **(C)** one-repetition maximum (1RM) squat strength before and after 8 weeks of plyometric training (PT) or traditional resistance training (RT). Data are presented as mean ± SD. PT, plyometric training group; RT, traditional resistance training group; CMJ, countermovement jump; RSI, reactive strength index; 1RM, one-repetition maximum. Significant group  ×  time interactions were observed for all three variables (*p* < 0.01). No significant between-group differences were detected at baseline or post-intervention for CMJ height and squat 1RM, whereas post-intervention RSI was significantly higher in PT than in RT (*p* = 0.007).

**Table 4:** Pre- and post-training values, within-group changes, and between-group differences in change (95% CI) for all outcome measures.

Variable	Group	Pre (mean ± SD)	Post (mean ± SD)	Within-group change	Between-group difference in change (PT − RT) (95% CI)	*p* (group difference)	Time × Group p	Partial *η*^2^
Squat 1RM (kg)	PT	89.42 ± 8.66	95.25 ± 7.83	+5.83	2.83 (1.37, 4.30)	0.001	0.001	0.423
	RT	87.75 ± 9.87	90.75 ± 8.82	+3.00				
Kick speed (m/s)	PT	11.10 ± 0.84	11.54 ± 0.72	+0.44	0.37 (0.25, 0.49)	<0.001	<0.001	0.661
	RT	10.93 ± 0.99	11.00 ± 0.97	+0.07				
Kick power (N)	PT	3,173.17 ± 159.31	3,315.92 ± 137.41	+142.75	84.17 (54.86, 113.48)	<0.001	<0.001	0.617
	RT	3,168.50 ± 176.41	3,227.08 ± 158.78	+58.58				
Kick repetitions (n/20s)	PT	14.75 ± 1.22	16.67 ± 0.89	+1.92	1.00 (0.33, 1.67)	0.005	0.005	0.303
	RT	14.67 ± 1.07	15.58 ± 0.90	+0.91				
CMJ height (cm)	PT	41.31 ± 1.33	43.03 ± 1.20	+1.72	1.06 (0.63, 1.49)	<0.001	<0.001	0.552
	RT	40.34 ± 4.83	40.99 ± 4.73	+0.65				
RSI	PT	1.34 ± 0.07	1.43 ± 0.05	+0.09	0.06 (0.04, 0.08)	<0.001	<0.001	0.706
	RT	1.34 ± 0.06	1.37 ± 0.05	+0.03				
Wingate peak power (W)	PT	736.42 ± 57.35	778.17 ± 47.13	+41.75	7.58 (−13.31, 28.48)	0.460	0.460	0.025
	RT	742.75 ± 57.64	776.92 ± 56.91	+34.17				
Wingate mean power (W)	PT	534.33 ± 46.78	599.17 ± 45.30	+64.83	38.50 (31.37, 45.63)	<0.001	<0.001	0.851
	RT	541.75 ± 56.17	568.08 ± 54.14	+26.33				

PT, plyometric training; RT, traditional resistance training; CMJ, countermovement jump; RSI, reactive strength index. Between-group difference in change was calculated as PT change minus RT change. *p* values were derived from independent-samples t-tests comparing change scores; for variables where Levene's test indicated unequal variances (kick speed, CMJ), the corrected t-test values are reported. Time  ×  Group *p* values are from two-way repeated-measures ANOVA. Partial *η*^2^ are reported for the group  ×  time interaction.

### 3.3 Kick performance

#### 3.3.1 Kick power

A significant group  ×  time interaction was detected for kick power (F₁,₂₂ = 35.464, *p* < 0.001, partial *η*^2^ = 0.617). As shown in Figure 3A, the PT group increased from 3,173.17 ± 159.31 N to 3,315.92 ± 137.41 N, with a mean change of +142.75 N (95% CI: 122.02 to 163.48 N), while the RT group increased from 3,168.50 ± 176.41 N to 3,227.08 ± 158.78 N, with a mean change of +58.58 N (95% CI: 37.86 to 79.31 N). Both groups improved significantly over time (both *p* < 0.001). No significant between-group difference was found at baseline (*p* = 0.946) or after training (*p* = 0.157). The significant group  ×  time interaction indicates that the magnitude of improvement was greater in the PT group than in the RT group (Table 4).

**Figure 3:**
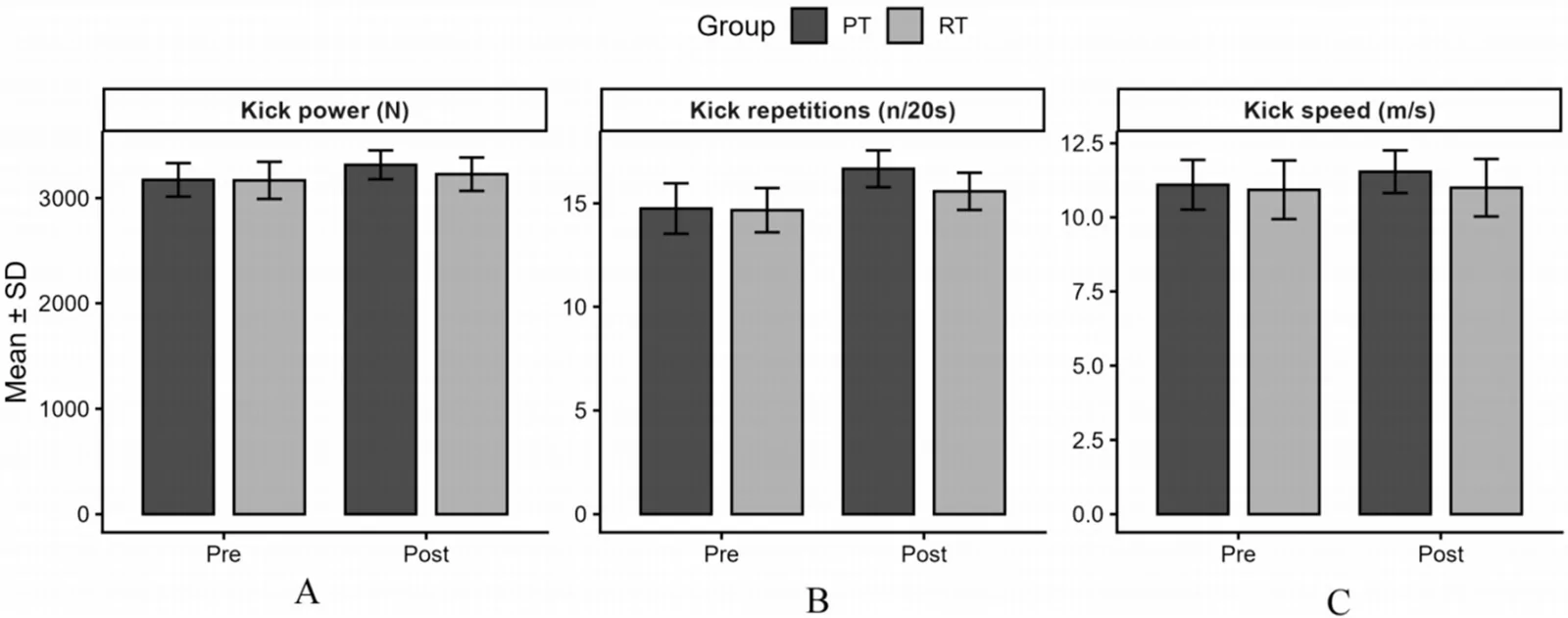
Changes in **(A)** roundhouse kick power, **(B)** 20-second continuous kick repetitions, and **(C)** roundhouse kick speed before and after 8 weeks of plyometric training (PT) or traditional resistance training (RT). Data are presented as mean ± SD. PT, plyometric training group; RT, traditional resistance training group. Significant group  ×  time interactions were observed for all three variables (*p* < 0.01). No significant between-group differences were detected at baseline or post-intervention for kick power and kick speed, whereas post-intervention kick repetitions were significantly higher in PT than in RT (*p* = 0.007).

#### 3.3.2 20-second continuous kick count

A significant group  ×  time interaction was observed for kick repetitions (F₁,₂₂ = 9.542, *p* = 0.005, partial *η*^2^ = 0.303). As illustrated in Figure 3B, the PT group increased from 14.75 ± 1.22 to 16.67 ± 0.89 repetitions, with a mean change of +1.92 reps (95% CI: 1.44 to 2.39 reps), while the RT group increased from 14.67 ± 1.07 to 15.58 ± 0.90 repetitions, with a mean change of +0.91 reps (95% CI: 0.44 to 1.39 reps). Both groups improved significantly (PT: *p* < 0.001; RT: *p* = 0.001). At baseline, no between-group difference was observed (*p* = 0.860). After training, however, the PT group had significantly higher kick repetitions than the RT group (*p* = 0.007), indicating a greater improvement in kick-specific speed endurance following plyometric training (Tables 4, 5).

**Table 5:** Simple effects analysis for outcomes with significant group  ×  time interactions.

Variable	Group	Pre vs. Post *p*	Between-group *p* at Post
Squat 1RM	PT	<0.001	0.200
	RT	<0.001	
Kick speed	PT	<0.001	0.135
	RT	0.088	
Kick power	PT	<0.001	0.157
	RT	<0.001	
Kick repetitions	PT	<0.001	0.007
	RT	0.001	
CMJ height	PT	<0.001	0.162
	RT	<0.001	
RSI	PT	<0.001	0.007
	RT	<0.001	
Wingate mean power	PT	<0.001	0.141
	RT	<0.001	

PT, plyometric training; RT, traditional resistance training. Between-group *p* at baseline was not significant for any variable (all *p* > 0.05). All *p* values for simple effects were adjusted using the Bonferroni correction.

#### 3.3.3 Kick speed

A significant group  ×  time interaction was found for kick speed (F₁,₂₂ = 42.989, *p* < 0.001, partial *η*^2^ = 0.661). As shown in Figure 3C, the PT group increased from 11.10 ± 0.84 m/s to 11.54 ± 0.72 m/s, with a mean change of +0.44 m/s (95% CI: 0.36 to 0.52 m/s), whereas the RT group showed minimal change, from 10.93 ± 0.99 m/s to 11.00 ± 0.97 m/s, with a mean change of +0.07 m/s (95% CI: −0.01 to 0.15 m/s), which was not statistically significant (*p* = 0.088). The improvement in the PT group was significant (*p* < 0.001). No significant between-group differences were observed at baseline (*p* = 0.649) or after training (*p* = 0.135). These findings indicate that plyometric training, but not traditional resistance training, produced a meaningful improvement in roundhouse kick speed (Table 4).

### 3.4 Explosive strength and reactive strength

#### 3.4.1 Countermovement jump height

A significant group  ×  time interaction was found for CMJ height (F₁,₂₂ = 27.138, *p* < 0.001, partial *η*^2^ = 0.552). As illustrated in Figure 2A, the PT group increased from 41.31 ± 1.33 cm to 43.03 ± 1.20 cm, with a mean change of +1.72 cm (95% CI: 1.42 to 2.02 cm), whereas the RT group increased from 40.34 ± 4.83 cm to 40.99 ± 4.73 cm, with a mean change of +0.65 cm (95% CI: 0.35 to 0.95 cm). Both groups improved significantly (both *p* < 0.001). There were no significant between-group differences at baseline (*p* = 0.507) or after training (*p* = 0.162), although the improvement was clearly larger in the PT group (Table 4).

#### 3.4.2 Reactive strength index

A significant group  ×  time interaction was detected for RSI (F₁,₂₂ = 52.898, *p* < 0.001, partial *η*^2^ = 0.706). As shown in Figure 2B, the PT group increased from 1.34 ± 0.07 to 1.43 ± 0.05, with a mean change of +0.09 (95% CI: 0.07 to 0.10), while the RT group increased from 1.34 ± 0.06 to 1.37 ± 0.05, with a mean change of +0.03 (95% CI: 0.01 to 0.04). Both groups improved significantly (both *p* < 0.001). At baseline, no significant difference was found (*p* = 1.000). After training, the PT group demonstrated significantly higher RSI than the RT group (*p* = 0.007), indicating a superior adaptation in stretch-shortening cycle function following plyometric training (Tables 4, 5).

### 3.5 Anaerobic power

#### 3.5.1 Wingate mean power

A significant group  ×  time interaction was found for Wingate mean power (F₁,₂₂ = 125.555, *p* < 0.001, partial *η*^2^ = 0.851). As illustrated in Figure 4A, the PT group increased from 534.33 ± 46.78 W to 599.17 ± 45.30 W, with a mean change of +64.83 W (95% CI: 59.80 to 69.87 W), while the RT group increased from 541.75 ± 56.17 W to 568.08 ± 54.14 W, with a mean change of +26.33 W (95% CI: 21.30 to 31.37 W). Both groups improved significantly (both *p* < 0.001). No significant between-group differences were found at baseline (*p* = 0.729) or after training (*p* = 0.141), although the PT group exhibited a markedly larger improvement (Table 4).

**Figure 4:**
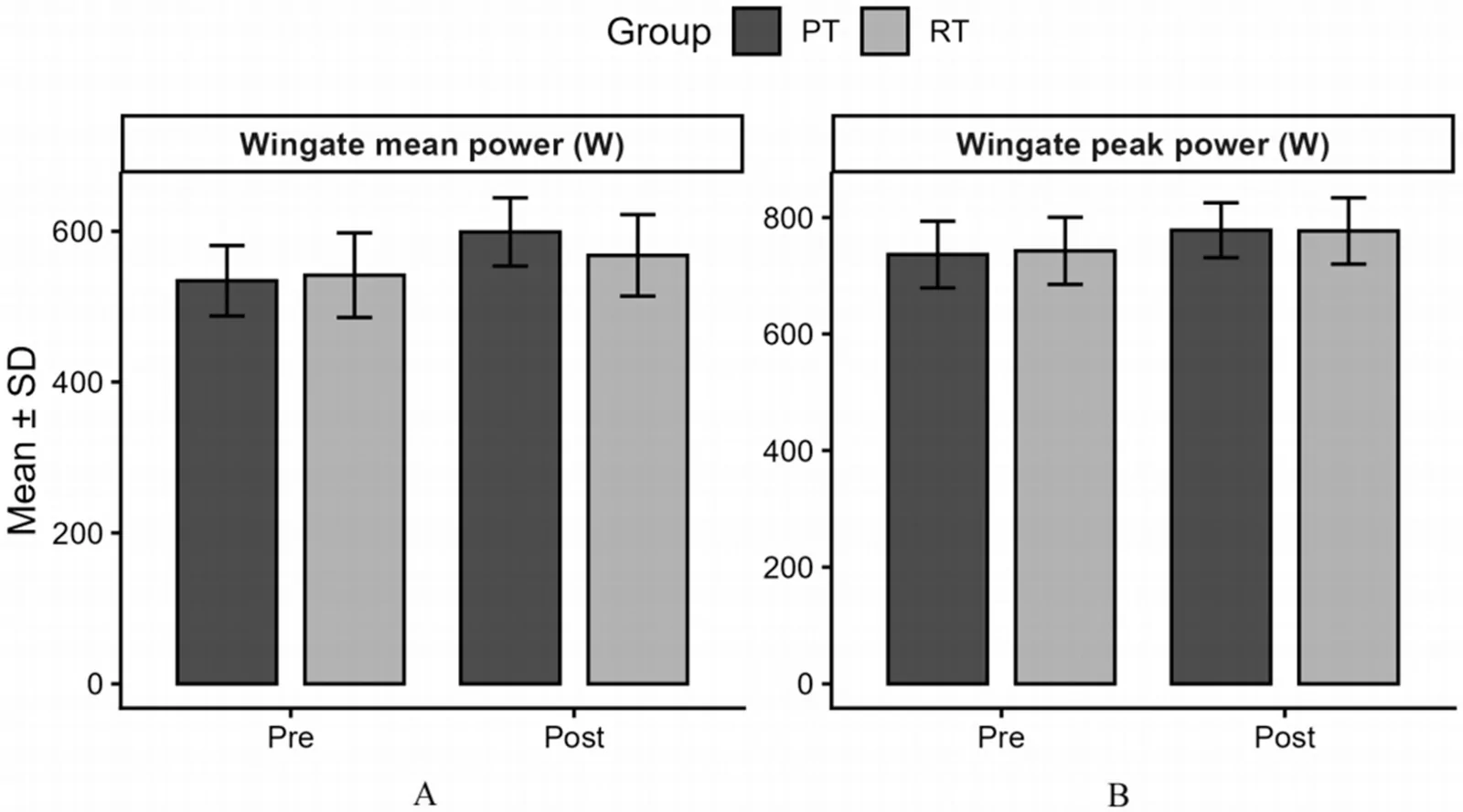
Changes in **(A)** Wingate mean power and **(B)** Wingate peak power before and after 8 weeks of plyometric training (PT) or traditional resistance training (RT). Data are presented as mean ± SD. PT, plyometric training group; RT, traditional resistance training group. A significant group  ×  time interaction was observed for Wingate mean power (*p* < 0.001), whereas no significant interaction was found for Wingate peak power (*p* = 0.460). Both groups improved significantly in both variables after training (all *p* < 0.001), with no significant between-group differences at post-intervention.

#### 3.5.2 Wingate peak power

No significant group  ×  time interaction was observed for Wingate peak power (F₁,₂₂ = 0.566, *p* = 0.460, partial *η*^2^ = 0.025). As shown in Figure 4B, the PT group increased from 736.42 ± 57.35 W to 778.17 ± 47.13 W, and the RT group increased from 742.75 ± 57.64 W to 776.92 ± 56.91 W. The main effect of time was significant (F₁,₂₂ = 56.757, *p* < 0.001, partial *η*^2^ = 0.721), and simple effects analysis confirmed that both groups improved significantly over time (PT: +41.75 W, 95% CI: 26.97 to 56.53 W, *p* < 0.001; RT: +34.17 W, 95% CI: 19.39 to 48.94 W, *p* < 0.001). Because the interaction was not significant, no between-group comparison was performed at individual time points (Table 4).

### 3.6 Effect size interpretation

According to Cohen's benchmarks, the group  ×  time interaction effects for squat 1RM, kick speed, kick power, CMJ height, RSI, and Wingate mean power were all large (partial *η*^2^ > 0.14). The interaction effect for Wingate peak power was negligible (partial *η*^2^ = 0.025), indicating similar improvements in both groups.

### 3.7 Summary of results

Overall, the PT group showed greater pre-to-post improvements than the RT group in squat 1RM, kick speed, kick power, kick repetitions, CMJ height, RSI, and Wingate mean power, as indicated by significant group  ×  time interactions. Wingate peak power increased similarly in both groups.

## 4 Discussion

### 4.1 Summary of main findings

This study examined the effects of 8 weeks of plyometric training and traditional resistance training on roundhouse kick performance, lower-body maximal strength, and anaerobic power in elite male taekwondo athletes. The principal finding was that plyometric training led to greater pre-to-post improvements than traditional resistance training in roundhouse kick speed, kick power, kick repetitions, CMJ height, reactive strength index, and Wingate mean power. The two interventions produced similar gains in Wingate peak power. In addition, squat 1RM improved significantly in both groups, with a slightly larger absolute increase in the plyometric training group, although the between-group difference was not statistically significant. Overall, these results support the hypothesis that explosive stretch-shortening cycle training produces greater improvements in high-velocity striking actions in taekwondo compared with heavy-load resistance exercise alone.

### 4.2 Roundhouse kick performance

The plyometric training group increased kick speed by approximately 4.0%, while the traditional resistance training group showed almost no change. This finding is consistent with previous studies demonstrating that ballistic and plyometric interventions are more effective than heavy resistance training for improving movement velocity in striking actions (19). The relatively small improvement in kick speed following resistance training is not surprising, because traditional slow-speed strength exercises mainly improve force production at low velocities, while kicking performance relies on force application within very short ground-contact and limb-acceleration phases. Similar dissociations between strength gains and speed improvements have been reported in boxing and taekwondo (20).

Kick power also showed a significant group-by-time interaction, with the plyometric training group improving by approximately 4.5% compared with only 1.8% in the resistance training group. Since kick power is the product of force and velocity, improvements in both the force-generating capacity and the velocity of limb movement are necessary. Plyometric training appears to target both components simultaneously by requiring high force output under rapid movement conditions. This interpretation is consistent with the training specificity literature, which suggests that high-velocity training produces broader adaptations across the force-velocity spectrum than heavy slow-speed training (21).

The 20-second continuous kick test revealed a significant post-intervention difference between groups, with the plyometric group improving by 13.0% and the resistance group by only 6.2%. This measure reflects not only single-kick ability but also the capacity to sustain repeated high-intensity efforts. The larger improvement in the plyometric group may partly result from improved intermuscular coordination and reduced unnecessary co-contraction during rapid alternating movements, allowing more efficient energy use across consecutive kicks (22). Because taekwondo competition involves repeated high-frequency kicking over multiple rounds, this improvement may have practical relevance beyond laboratory measures.

### 4.3 Lower-body strength and explosive power

CMJ height improved by 4.2% in the plyometric training group but only 1.6% in the traditional resistance training group. This is consistent with a large body of evidence showing that plyometric training is one of the most effective methods for improving vertical jump performance (11). The mechanism is generally attributed to enhanced use of the stretch-shortening cycle, increased musculotendinous stiffness, and improved neural drive during explosive muscle actions. The present data extend these findings to elite taekwondo athletes, a population that has received relatively little attention in this context.

Reactive strength index improved significantly in both groups, but the improvement was approximately three times larger in the plyometric group than in the resistance group, and the post-intervention between-group difference was significant. Because RSI reflects the ability to absorb and rapidly reapply force during ground contact, it is considered a key indicator of stretch-shortening cycle function. The large interaction effect observed here indicates that plyometric training produced a more specific adaptation in fast force reversal. Given that taekwondo kicking requires the support leg to quickly transition from yielding to propulsion, improved reactive strength may be one of the primary mechanisms through which plyometric training enhanced kick speed and power (23).

The greater improvement in squat 1RM in the PT group was unexpected, as heavy resistance exercise is usually considered the most effective method for increasing maximal strength. One possible explanation is that the high-impact plyometric exercises provided a strong neural stimulus for the lower limbs, which may have improved maximal force production in athletes with relatively modest baseline strength. Another possibility is that the RT group had more previous experience with heavy resistance training, and therefore their strength gains were closer to a plateau. However, because the between-group difference at post-intervention was not significant, this finding should be interpreted with caution and should not be used to suggest that plyometric training is superior to resistance training for maximal strength development. Previous studies have generally found that resistance training produces larger 1RM gains than plyometric training, especially in stronger or more experienced athletes (24).

### 4.4 Anaerobic power

The pattern of results for the Wingate test provides further insight into the nature of the training adaptations. Wingate peak power increased significantly and similarly in both groups, suggesting that both training modes improved maximal anaerobic power to a comparable extent. Because peak power in the Wingate test is strongly influenced by maximal neuromuscular power and phosphocreatine utilization, it is not surprising that both interventions led to improvements in this measure. Heavy resistance training has been shown to increase lower-limb maximal power, particularly when combined with the intention to move loads explosively (25).

In contrast, Wingate mean power showed a large and significant group-by-time interaction, with the plyometric group improving by 12.1% compared with only 4.9% in the resistance group. Mean power during a 30-second maximal cycling test depends not only on peak power but also on the ability to sustain energy production through anaerobic glycolysis and to resist fatigue. The larger improvement in the plyometric group may reflect better maintenance of high-intensity output, but this cannot be confirmed because physiological variables such as muscle buffering capacity were not measured in the present study. The 20-second continuous kick test results partially support the idea that plyometric training improved sport-specific repeated-effort capacity, although the mechanisms may differ between cycling and kicking.

### 4.5 Comparison with previous studies

Previous research in combat sports has shown that plyometric training improves kicking speed and power in taekwondo and karate athletes (26). However, many of those studies used recreational or lower-level athletes, which limits their applicability to high-level competitors. The present findings extend this work by demonstrating that elite taekwondo athletes can still achieve meaningful improvements in kick performance after a structured plyometric intervention. A direct comparison with previous studies is difficult because of differences in test protocols, intervention duration, and outcome measures, but the magnitude of improvement observed here is broadly comparable to reports from other striking-based sports.

### 4.6 Limitations

Several limitations should be acknowledged when interpreting the present findings.

(1) Sample characteristics and generalizability. The participants were 24 elite male taekwondo athletes recruited from a single university team, with body mass restricted to 58–68 kg. Although this improved homogeneity, the findings may not extend to female athletes, heavier weight categories, or higher-level competitors from different training systems.
(2) Absence of a non-training control group. The design did not include a control group that continued only regular taekwondo training without additional lower-body conditioning. Therefore, the possibility that part of the observed improvements resulted from familiarization with the testing procedures or from concurrent sport-specific training cannot be fully excluded, although the randomized allocation and significant group-by-time interactions reduce this concern.
(3) Inability to match total training load between interventions. The two programs differed fundamentally in movement pattern, contraction velocity, and mechanical loading. Although session RPE was recorded and was comparable between groups, total mechanical work and neuromuscular stress could not be precisely equated, which may have contributed to the different magnitudes of adaptation.
(4) Short intervention period and laboratory-based performance measures. The 8-week training duration cannot address long-term adaptations or whether the between-group differences would persist or increase over a longer period. In addition, kick performance was assessed under standardized laboratory conditions, which may not fully reflect the speed, force, and repeated-kick demands of official competition.

### 4.7 Practical applications

From a practical perspective, these results support the inclusion of a progressive plyometric training block in the physical preparation of taekwondo athletes. When the training priority is to enhance kick speed, kick power, or repeated kicking ability, plyometric exercises appear to be more effective than traditional heavy resistance training alone. That said, traditional resistance training remains important for maintaining maximal strength and for providing the base upon which explosive capacities are built. A combined approach that uses heavy resistance exercise to develop maximal force and plyometric exercise to develop high-speed force application would likely produce the most complete adaptations.

The training program used in the present study was relatively simple and required minimal equipment, which supports its feasibility in applied sport settings. Coaches should note that plyometric training must be introduced progressively and performed with adequate technical quality. In this study, the intensity and complexity of exercises were gradually increased across four two-week phases, and no injuries occurred. This model may be useful for teams that have limited access to specialized equipment.

## 5 Conclusions

The present study demonstrated that 8 weeks of plyometric training resulted in greater pre-to-post improvements in roundhouse kick speed, kick power, repeated kicking ability, countermovement jump height, reactive strength index, and Wingate mean power compared with traditional resistance training in elite male taekwondo athletes. Both training modes produced similar improvements in Wingate peak power, and although the plyometric group showed a larger increase in squat 1RM, the post-intervention difference between groups did not reach statistical significance.

These findings should not be interpreted as evidence that plyometric training is universally superior, because several outcomes did not differ significantly between groups at post-intervention. The term “transfer” was not directly assessed in this study and should be interpreted with caution. From a practical perspective, a progressive plyometric program can be effectively integrated into the physical preparation of taekwondo athletes when the primary goal is to enhance kick speed, kick power, or repeated kicking capacity. Traditional resistance training should not be discarded, as it remains important for developing maximal strength, but coaches should consider that heavy slow-speed training may have limited direct transfer to fast kicking movements.

Future studies should examine whether combining plyometric and traditional resistance training produces superior adaptations compared with either method alone. Longer intervention periods, larger samples, and more ecologically valid kick performance assessments under competitive conditions would also strengthen the evidence base.

## Data Availability

The original contributions presented in the study are included in the article/Supplementary Material, further inquiries can be directed to the corresponding author.
